# Evaluation of Tumor DNA Sequencing Results in Patients with Gastric and Gastroesophageal Junction Adenocarcinoma Stratified by *TP53* Mutation Status

**DOI:** 10.1093/oncolo/oyac018

**Published:** 2022-02-26

**Authors:** Anthony C Wood, Yonghong Zhang, Qianxing Mo, Ling Cen, Jacques Fontaine, Sarah E Hoffe, Jessica Frakes, Sean P Dineen, Jose M Pimiento, Christine M Walko, Rutika Mehta

**Affiliations:** 1 Department of Gastrointestinal Oncology, Moffitt Cancer Center & Research Institute, Tampa, FL, USA; 2 Department of Biostatistics and Bioinformatics, Moffitt Cancer Center & Research Institute, Tampa, FL, USA; 3 Department of Thoracic Oncology, Moffitt Cancer Center & Research Institute, Tampa, FL, USA; 4 Department of Radiation Oncology, Moffitt Cancer Center & Research Institute, Tampa, FL, USA; 5 Department of Individualized Cancer Medicine, Moffitt Cancer Center & Research Institute, Tampa, FL, USA

**Keywords:** DNA sequencing, TP53, gastric cancer, gastroesophageal cancer, mutation analysis

## Abstract

**Background:**

Gastric cancer (GC) and gastroesophageal junction adenocarcinomas (GEJ) are molecularly diverse. *TP53* is the most frequently altered gene with approximately 50% of patients harboring mutations. This qualitative study describes the distinct genomic alterations in GCs and GEJs stratified by *TP53* mutation status.

**Patients and Methods:**

Tumor DNA sequencing results of 324 genes from 3741 patients with GC and GEJ were obtained from Foundation Medicine. Association between gene mutation frequency and *TP53* mutation status was examined using Fisher’s exact test. Functional gene groupings representing molecular pathways suggested to be differentially mutated in *TP53* wild-type (*TP53*_*WT*_) and *TP53* mutant (*TP53*_*MUT*_) tumors were identified. The association of the frequency of tumors containing a gene mutation in the molecular pathways of interest and *TP53* mutation status was assessed using Fisher’s exact test with a *P*-value of <.01 deemed statistically significant for all analyses.

**Results:**

*TP53* mutations were noted in 61.6% of 2946 GCs and 81.4% of 795 GEJs (*P* < .001). Forty-nine genes had statistically different mutation frequencies in *TP53*_*WT*_ vs. *TP53*_*MUT*_ patients. *TP53*_WT_ tumors more likely had mutations related to DNA mismatch repair, homologous recombination repair, DNA and histone methylation, Wnt/B-catenin, PI3K/Akt/mTOR, and chromatin remodeling complexes. *TP53*_*MUT*_ tumors more likely had mutations related to fibroblast growth factor, epidermal growth factor receptor, other receptor tyrosine kinases, and cyclin and cyclin-dependent kinases.

**Conclusion:**

The mutational profiles of GCs and GEJs varied according to *TP53* mutation status. These mutational differences can be used when designing future studies assessing the predictive ability of *TP53* mutation status when targeting differentially affected molecular pathways.

## Teaser

Gastric (GC) and gastroesophageal junction adenocarcinomas (GEJ) are molecularly diverse. TP53 is the most frequently altered gene, with approximately 50% of patients harboring mutations. This article describes the distinct genomic alterations in GCs and GEJs stratified by TP53 mutation status.

**Implications for Practice**

Despite being mutated in approximately 50% of gastroesophageal adenocarcinomas (GEAs), therapeutic interventions taking *TP53* mutation status into account in a predictive capacity have yet to be developed. Additionally, there is limited information available assessing its ability to function as a predictive marker in the era of targeted cancer therapy. The mutational differences of GEAs stratified by *TP53* mutation status as described in this manuscript can be used as a tool when conceiving future pre-clinical and clinical studies to assess the predictive ability of *TP53* mutational status when using molecularly targeted agents.

## Introduction

Gastric and esophageal cancer are the third and sixth leading cause of cancer deaths worldwide.^1^ In the US alone, 26 250 new cases of gastric cancer and 19 260 new cases of esophageal cancer were diagnosed in 2021, with males representing 61% and 79% of these new diagnoses.^2^ The western population, in particular, has seen the incidence of gastric cancer (GC) and steady increase of gastroesophageal junction adenocarcinomas (GEJs) due to the increasing prevalence of obesity and gastro-esophageal reflux disease.^3-5^ GCs and GEJs, collectively known as gastroesophageal adenocarcinomas (GEAs), are molecularly diverse. Attempts have been made to molecularly categorize these tumors to organize future studies and create tailored treatment strategies. In 2014, the Cancer Genome Atlas Research Network (TCGA) analyzed 295 GC samples using array-based somatic copy number analysis, whole-exome sequencing, array-based DNA methylation profiling, messenger RNA (mRNA) sequencing, microRNA (miRNA) sequencing, reverse-phase protein array, and microsatellite instability (MSI) testing to propose separating GC into 4 general molecular subtypes.^6^ The first 2 molecular subtypes were generated on the basis of CpG island hypermethylation (CIMP): Epstein-Barr virus (EBV)+ CIMP tumors (9%) with distinct hypermethylation patterns not associated with the epigenetic silencing of *MLH1*, and microsatellite instability (MSI) enriched CIMP EBV-tumors (22%) with hypermethylation patterns consistent with *MLH1* epigenetic silencing. The remainder of the GCs were then stratified into one of 2 groups based on their relative burden of somatic copy number aberrations (SCNAs): genomically stable (GS) tumors (20%) had less SCNAs as compared to those deemed to have chromosomal instability (CIN, 50%).^6^

Commonly occurring genetic mutations in GEAs include *ARID1A*, *PIK3CA*, *KRAS*, and *CDH1*.^7^ However, *TP53* is the most frequently altered gene with approximately 50% of GEAs and 70% of CIN GCs harboring mutations.^6,8^*TP53* encodes the tumor suppressor p53 protein that functions as a key member of the G1/S checkpoint to help maintain genetic integrity through the cell cycle.^9^ In times of cellular stress *TP53* expression increases to mediate G1 phase cell cycle arrest to facilitate repair before cellular division or promote apoptosis in cells with an overabundance of molecular derangements that cannot be overcome. When there is a loss of *TP53* function, the G1/S checkpoint is effectively lost, and genetically damaged cells have significantly less barriers to unchecked proliferation with negative physiologic consequences. Therefore, it is not surprising that inactivating *TP53* mutations have been implicated in the carcinogenesis of a multitude of malignancies, with GEAs being no exception.^10^ In fact, the Asian Cancer Research Group (ACRG) included *TP53* expression as part of their proposed molecular classification of GC.^11^ Using gene expression panels derived from 300 GCs, the ACRG first separated GCs molecularly into 2 large categories: MSI and microsatellite stable (MSS). MSS tumors were then stratified by the presence of, or lack thereof, an epithelial-to-mesenchymal gene signature (MSS/EMT). The remaining MSS/EMT^-^ tumors were divided into the final 2 categories, MSS*/TP53*^*+*^ and MSS/*TP53*^−^, based on levels of *TP53* expression with noted differences in patient characteristics between these 2 groups.^11^

Next-generation sequencing (NGS) is now widely used for most advanced cancers as an aid to elucidate the genomic characteristics of tumors. While therapeutic interventions for GEAs taking *TP53* mutation status into account have yet to be developed, its high prevalence makes it a subject of interest to clinical investigators as a potential predictive marker.^4,10,12-14^ An understanding of commonly co-occurring genetic alterations in *TP53* wild-type (*TP53*_*WT*_) and *TP53* mutated (*TP53*_*MUT*_) GEAs may help guide clinically practical investigations in the future assessing this potential. This study identified the unique mutational profiles of *TP53*_*WT*_ and *TP53*_*MUT*_ GEAs by analyzing the DNA sequencing results of 3741 GEA tumor samples.

## Patients and Methods

De-identified data were acquired from Foundation Medicine for all available patients with GEA for whom the FoundationOne CDx (F1CDx) assay was performed within the US. F1CDx is a commercially available NGS diagnostic test developed by Foundation Medicine that uses targeted high throughput hybridization-based capture technology for the detection of substitutions, insertion and deletion alterations, and copy number alterations in 324 genes and select gene rearrangements using DNA isolated from formalin-fixed paraffin-embedded tumor tissue specimens.^15^ The data provided included age at the time of the assay results, gender, tumor mutational burden (TMB), and the distinct genomic alterations noted on DNA sequencing. Information on MSI and PD-L1 status was not available in the dataset. The dataset was sorted by *TP53* mutation status. Differences in mutation frequency were detected using Fisher’s exact test of independence with a *P*-value of <.01 designated as the cutoff value for statistical significance. Statistical significance for continuous data was tested using a 2 samples *t*-test for mean values and the Mann-Whitney U test for distribution/median values.

Pathway analysis was completed by first isolating the genes with statistically different mutation frequencies between *TP53*_*WT*_ and *TP53*_*MUT*_ tumors. After studying these results, 12 predetermined functional gene groupings were created to help elucidate molecular pathways suggested to be differentially affected in *TP53*_*WT*_ and *TP53*_*MUT*_ tumors. The number of tumors containing a mutation in at least one of the prespecified genes in the molecular pathways of interest was counted. Then, the association of the frequency of tumors containing a gene mutation in the molecular pathways of interest and *TP53* mutation status was assessed using Fisher’s exact test of independence with a *P*-value of <.01 deemed statistically significant.

## Results

### Demographics

The dataset consisted of 3741 patients with GEA: 2946 GCs and 795 GEJs. The entire study population was 65.2% male and 34.8% female. GC patients were 60.6% male and 39.4% female, whereas patients with GEJ were 82.4% male and 17.6% female (*P* < .001). The median patient age was 63, which was similar between *TP53*_*WT*_ and *TP53*_*MUT*_ patients.

### Mutations Stratified by TP53 Mutation Status

**Figure 1.**
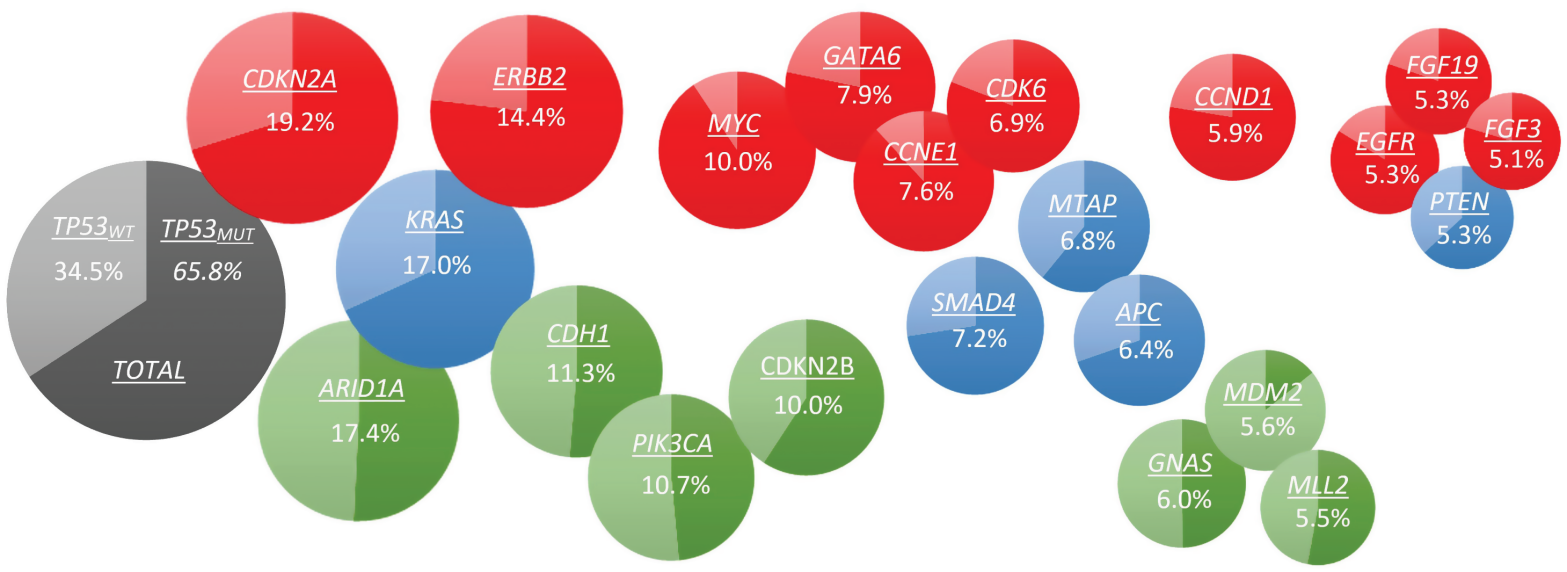
Compound bubble pie chart of the 22 genes with *>*5% gene mutation frequency within the entire dataset (GEAs) in order of decreasing frequency. Each pie chart represents an individual gene broken down by the relative frequency in which patients harbor a mutation stratified by *TP53* mutation status: the lighter color represents *TP53*_*WT*_ patients, and the darker color represents *TP53*_*MUT*_ patients. The numbers within the pie charts describe the frequency of the gene mutation within the entire dataset. Statistical significance of mutation frequency between *TP53*_*WT*_ and *TP53*_MUT_ tumors is designated using a color-coded scheme. Green: *TP53*_*MUT*_ < *TP53*_*WT*_. Red: *TP53*_*MUT*_ > *TP53*_*WT*_. Blue: *TP53*_*MUT*_ = *TP53*_*WT*_.

**Figure 2.**
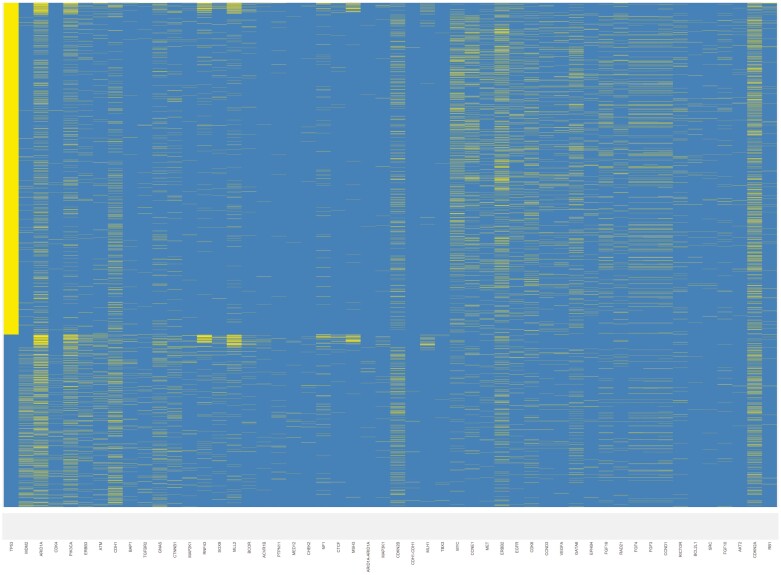
Distribution of gene mutations on an individual basis. Each row represents an individual. Each column represents one of the 49 genes noted to be differentially mutated stratified by *TP53* mutation status. The yellow tick mark designates that the patient contains a mutation in the gene of interest. The background blue color denotes wild-type status. The 49 gene names, in order by column, are as follows: TP53, MDM2, ARID1A, CDK4, PIK3CA, ERBB3, ATM, CDH1, BAP1, TGFBR2, GNAS, CTNNB1, MAP2K1, RNF43, SOX9, MLL2, BCOR, ACVR1B, PTPN11, MED12, CHEK2, NF1, CTCF, MSH3, ARID1A-ARID1A, MAP3K1, CDKN2B, CDH1-CDH1, MLH1, TBX3, MYC, CCNE1, MET, ERBB2, EGFR, CDK6, CCND3, VEGFA, GATA6, EPHB4, FGF19, RAD21, FGF4, FGF3, CCND1, RICTOR, BCL2L1, SRC, FGF10, AKT2, CDKN2A, RB1.

*TP53* mutations were present in 65.8% of specimens. *TP53* mutations were noted in 61.6% of GCs and 81.4% of GEJs (*P* < .001). The median TMB score was lower in *TP53*_*WT*_ as compared to *TP53*_*MUT*_ GEAs (2.5 vs. 3.8, *P* < .001). The frequency of tumors with a TMB score *>*10 mutations/megabase (11.1% vs. 9.4%; *P* = .11) were similar in *TP53*_*WT*_ and *TP53*_*MUT*_ groups, respectively. The most commonly mutated genes in the entire population other than *TP53* were *CDKN2A* (19.1%)*, ARID1A* (17.4%)*, KRAS* (17.0%)*, ERBB2* (14.4%), and *CDH1* (11.3%) (Fig. 1). Forty-nine of the 324 genes in the panel (15.1%) had statistically different mutation frequencies in *TP53*_*WT*_ vs. *TP53*_*MUT*_ patients (Fig. 2). The genetic alterations with the greatest over-representation in *TP53*_*WT*_ patients included amplification mutations of *MDM2* and *CDK4,* truncating mutations of *ARID1A,* point mutations of *PIK3CA,* and amplification/point mutations of *ERBB3* (Table 1). Amplification mutations of *MYC*, *CCNE1,* and *MET,* along with amplification/point mutations of *EGRF* and *ERBB2,* were the most over-represented genetic alterations in *TP53*_*MUT*_ patients (Table 2).

**Table 1.** Gene mutations overrepresented in *TP53*_*WT*_ GEAs.

Gene	TP53MUT (%)	TP53WT (%)	−log10(*P*-value)
*MDM2*	1.2	14.1	56.2
*ARID1A*	13.4	25.2	17.8
*CDK4*	0.4	3.9	14.4
*PIK3CA*	7.9	16.2	13.3
*ERBB3*	2.1	7	12.5
*ATM*	1.7	6	11
*CDH1*	8.8	16	10
*BAP1*	0.7	3.6	9.5
*TGFBR2*	0.6	2.9	7.5
*GNAS*	4.5	8.8	6.5
*CTNNB1*	2.8	6.1	5.5
*MAP2K1*	0.8	2.7	5.4
*RNF43*	3	6.3	5.2
*SOX9*	1.5	3.8	5.1
*MLL2*	4.4	7.6	4
*BCOR*	1.1	3	4
*ACVR1B*	0.3	1.4	3.5
*PTPN11*	0.5	1.7	3.4
*MED12*	0.1	0.8	3.2
*CHEK2*	0.3	1.2	2.5
*NF1*	2.2	3.8	2.4
*CTCF*	0.5	1.5	2.4
*MSH3*	1.4	2.7	2.3
*MAP3K1*	0.5	1.4	2.3
*CDKN2B*	9	11.9	2.2
*MLH1*	0.8	1.8	2.1
*TBX3*	0.1	0.5	2

**Table 2.** Gene mutations overrepresented in *TP53*_*MUT*_ GEAs.

Gene	TP53MUT (%)	TP53WT (%)	−log10(*P*-value)
*MYC*	13.8	2.8	30.4
*CCNE1*	10.2	2.7	18.2
*MET*	6.1	1.8	9.6
*ERBB2*	16.8	9.7	8.6
*EGFR*	6.8	2.5	8.3
*CDK6*	8.5	3.9	7.1
*CCND3*	4.8	1.6	6.7
*VEGFA*	4.8	1.6	6.3
*GATA6*	9.3	5	5.8
*EPHB4*	3.7	1.2	5.6
*FGF19*	6.5	3	5.3
*RAD21*	4.8	2	5.2
*FGF4*	6.1	2.8	5.1
*FGF3*	6.2	3	4.7
*CCND1*	6.9	3.8	4
*RICTOR*	3.9	1.6	3.9
*BCL2L1*	1.4	0.2	3.4
*SRC*	1.3	0.3	2.8
*FGF10*	2.3	0.9	2.5
*AKT2*	1.1	0.2	2.5
*CDKN2A*	20.5	16.8	2.2
*RB1*	1.9	0.8	2

### Pathway Analysis

**Figure 3.**
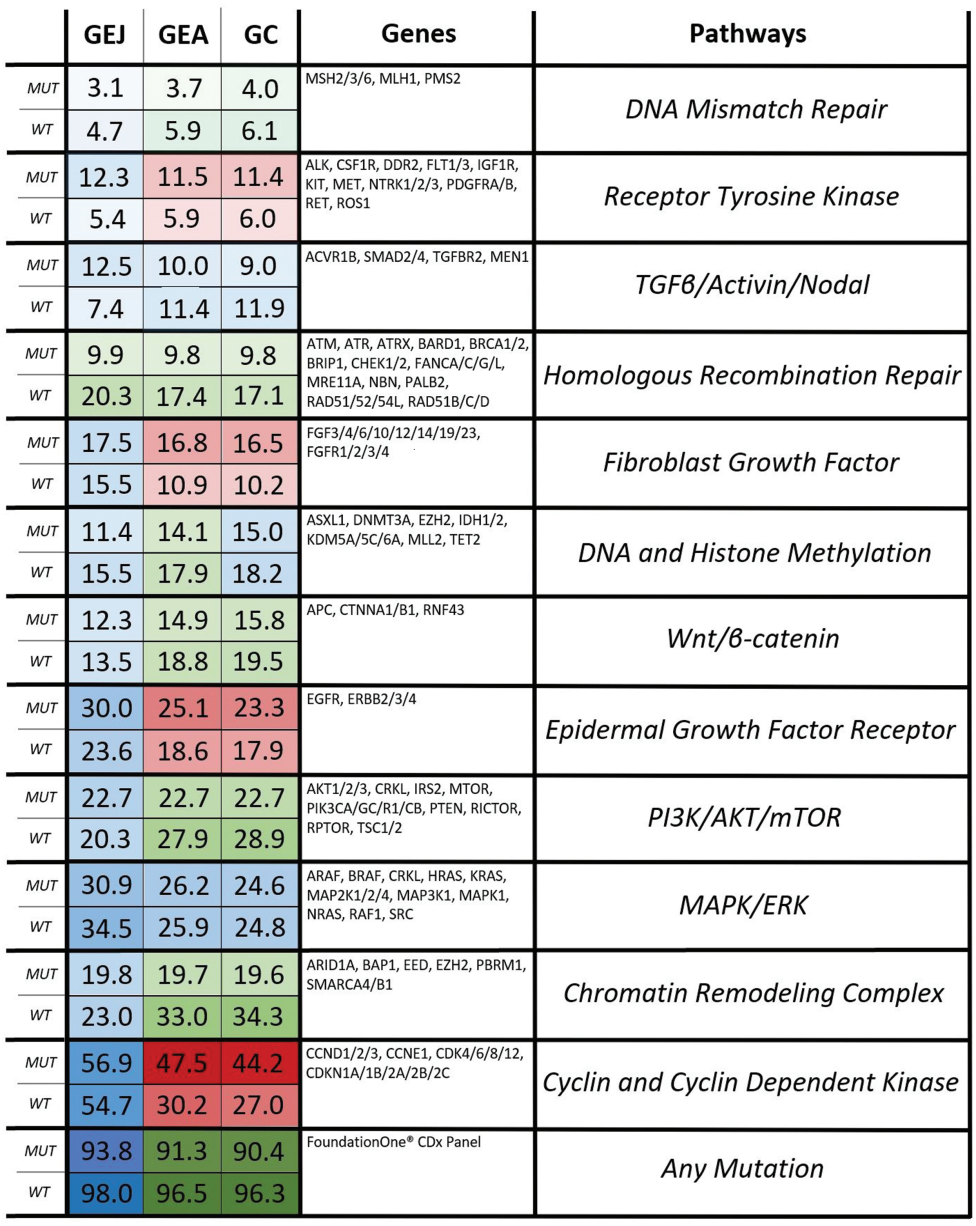
The final pathway analysis results. The column on the right describes the 12 functional gene groupings that were created to represent molecular pathways suggested to be differentially affected in *TP53*_*WT*_ and *TP53*_*MUT*_ GEAs. The purpose of this figure is to illustrate whether the mutational involvement of these pathways/gene groupings varied between *TP53*_*WT*_ and *TP53*_*MUT*_ GEAs (entire dataset), GCs, and GEJs. The column in the middle contains the pre-specified genes within the pathways of interest. The numbers in the left column describe the percentage of patients containing a mutation in at least one of the genes in the pathway of interest. The left column is broken down into 3 sub-columns by tumor type. Each row is broken down into 2 sub-rows denoting *TP53* mutation status. Statistical significance of mutation frequency between *TP53*_*WT*_ and *TP53*_MUT_ tumors is designated using a color-coded scheme. Green: *TP53*_*MUT*_ < *TP53*_*WT*_. Red: *TP53*_*MUT*_ > *TP53*_*WT*_. Blue: *TP53*_*MUT*_ = *TP53*_*WT*_.

The pathway analysis results are represented in Fig. 3. *TP53*_*WT*_ GEAs were more likely to contain a mutation in a gene related to DNA mismatch repair, homologous recombination repair, DNA and histone methylation, Wnt/B-catenin, PI3K/Akt/mTOR, and chromatin remodeling complexes. Furthermore, *TP53*_*WT*_ tumors were more likely to contain any mutation in the gene panel as compared to *TP53*_*MUT*_ tumors. *TP53*_*MUT*_ GEAs were more likely to contain a mutation in a gene related to fibroblast growth factor, epidermal growth factor receptor, other receptor tyrosine kinases, and cyclin and cyclin-dependent kinases. When broken down by tumor type, GCs had pathway analyses results that closely mirrored those of the entire dataset with the only difference being that the frequency of mutations in DNA and histone methylation genes was no longer statistically different between *TP53*_*WT*_ and *TP53*_*MUT*_ GCs (*P* = .026). On the other hand, the lone pathway that was differentially affected in *TP53*_*WT*_ and *TP53*_*MUT*_ GEJs was homologous recombination repair (20.3% vs. 9.9%, *P* = .001).

## Discussion

The most common predictive biomarkers currently used in GEAs are microsatellite instability or mismatch repair deficiency status and expression of human epidermal growth factor receptor 2 (HER2) and programmed death-ligand 1 (PD-L1).^16,17^ There are other biomarkers that will soon have therapeutic implications, such as Claudin 18.2 and FGFR2b.^18,19^ The use of these predictive biomarkers has helped us tailor treatments for patients with GEA, yet there is still an unmet need to identify additional targets for novel treatment strategies. In our study, we were able to demonstrate that the mutational profiles of GEAs differed when stratified according to *TP53* mutation status. *TP53* mutations were identified at higher rates in our sample of GEAs as compared to those described by TCGA and other manuscripts reporting such data.^4,8,20,21^ Genes directly involved in the DNA damage response and epigenetic regulation of DNA were more frequently mutated in *TP53*_*WT*_ tumors. Meanwhile, growth factor and receptor tyrosine kinase genes were more frequently mutated in *TP53*_*MUT*_ tumors.

GEJ tumors were more likely to be *TP53*_*MUT*_ than their GC counterparts (81.4% vs. 61.6%, *P*-value = <.001) in this dataset, which is consistent with DNA sequencing results previously described.^22,23^ Given this variability, there was interest in comparing GEJ and GC mutation profiles against one another to assess any further genetic differences stratified by *TP53* mutational status that may distinguish the 2 GEA malignancies. Homologous recombination repair genes were more frequently mutated in both *TP53*_*WT*_ GEJs and GCs. Additionally, the ratio of mutational involvement of DNA mismatch repair, DNA and histone methylation, epidermal growth factor, and receptor tyrosine kinase genes appeared to be consistent in GEJs and GCs when broken down by *TP53* mutation status. The remaining functional gene groupings, however, did not seem to have as much mutational variation between *TP53*_*WT*_ and *TP53*_MUT_ tumors in GEJs as compared to GCs. This can be most readily seen when looking at the involvement of cyclin and cyclin-dependent kinase genes, which play an integral role in the maintenance of the cell cycle in concert with *TP53.* Twenty-seven percent vs. 44.2% of *TP53*_*WT*_ and *TP53*_*MUT*_ GCs had cyclin and cyclin-dependent kinase gene mutations. Meanwhile, 54.7% vs. 56.9% of *TP53*_*WT*_ and *TP53*_MUT_ GEJs had cyclin and cyclin-dependent kinase gene mutations. On a gene-specific level, *ZNF217* (15.5%) and *AURKA* (8.1%) amplification mutations were disproportionately seen in *TP53*_*WT*_ GEJs as compared to the rest of the dataset (4.4% and 2.1%, respectively). As both *ZNF217* and *AURKA* genes are located on chromosome 20q13, it stands to reason that 20q13 amplification may play a prominent role in the carcinogenesis of *TP53*_*WT*_ GEJs.^20,24^ These findings suggest that despite their anatomic proximity the genetic makeup of GEJs and GCs are distinct from one another.

Studies assessing the prognostic impact of *TP53* mutations on the outcomes of GEAs have demonstrated conflicting results.^25-29^ The molecular characterization of GEAs in relation to TCGA subtype, co-occurring mutations, and the type of *TP53* mutation itself all seem to influence *TP53*’s prognostic significance.^11,12,30^ Equally, the ability of *TP53* mutation status to function as a predictive marker of treatment response is also unknown. A meta-analysis of 13 studies including 564 cases concluded that GCs with high levels of p53 expression on immunohistochemistry (IHC) have a decreased response to chemotherapy.^31^ Unfortunately, the correlation of p53 expression on IHC with *TP53* mutational status is not consistent, making it difficult to extrapolate the findings of this analysis toward tumor DNA sequencing results.^32,33^ More recent studies have attempted to delineate the predictive potential of *TP53* mutations in the era of molecularly targeted therapies and immunotherapies. One manuscript supporting its predictive ability in targeting the vascular endothelial growth factor (*VEGF)* pathway was recently published.^34^ In 48 patients with GC who received second-line ramicurimab/paclitaxel combination therapy the median OS was 9.5 months for carriers of *TP53*_*Inactive*_ mutations, 8.6 months for carriers of other *TP53* mutations, 6.0 months for carriers of *TP53*_*Active*_ missense mutations, and 4.5 months for *TP53*_*WT*_ patients (*P* = .01). *VEGFA* was accordingly more mutated in our group of *TP53*_*MUT*_ GEAs as compared to *TP53*_*WT*_ GEAs (4.8% vs. 1.6%, −log_10_(*P*-value) = 6.3). In another review of 356 GCs from the TCGA database, it was found that *TP53*_*MUT*_ tumors had lower levels of anti-tumor immunity and a decreased response to immune checkpoint inhibitor therapy as compared to *TP53*_*WT*_ tumors.^35^ On the other hand, sub-group analysis of the phase III GOLD trial investigating olaparib and paclitaxel combination therapy in recurrent and metastatic GC found no difference in therapeutic response based on *TP53* mutation status.^36^ More studies are needed to assess the predictive ability of *TP53* mutation in isolation and in conjunction with other molecular alterations.

The limitations of this study include missing data regarding clinical outcomes, microsatellite instability status, expression of other protein biomarkers, and additional patient characteristics necessary to organize these tumors into one of the 4 molecular subtypes as proposed by the Cancer Genome Atlas Research Network. In addition, while tissue-based assays offer a comprehensive genomic profile, there can be intra- and intertumoral (primary vs. metastatic site) heterogeneity which may not be fully encompassed on tissue NGS.^37^ This potentially limits the clinical utility of tissue-based mutation analysis as the full mutational landscape of the patient’s tumors may not be elucidated due to the sampling limitations inherent with this method of genetic profiling. This notion, along with the invasiveness of obtaining tumor samples for molecular analysis, has led to the increased use of liquid biopsies assessing circulating tumor DNA (ctDNA) NGS results in conjunction with tissue NGS to guide clinical decision-making. Nonetheless, as tissue-based DNA/RNA sequencing results yet remain standard in clinical practice, the observations of this study could serve as a foundation for practical future preclinical and clinical investigations that can help elucidate the clinical correlations of our findings. For example, genes in the PI3K/AKT/mTOR pathway were significantly more mutated in *TP53*_*WT*_ tumors, which was largely driven by the greater presence of *PIK3CA* mutations in *TP53*_*WT*_ as compared to *TP53*_*MUT*_ tumors (16.2% vs. 7.9%). Trials are currently underway assessing the role of PI3K inhibitors for *PIK3CA* mutated tumors in GEAs. It would be useful to perform a subgroup analysis of the results of these trials stratified by *TP53* mutation status to determine if these findings signal a pathophysiologic difference that carries predictive relevance in the real-world setting. Similar analyses could be completed for additional studies investigating therapeutic interventions targeting genes and cellular products related to the DNA damage response, epigenetic regulation of DNA, growth factors, receptor tyrosine kinases, cyclins and cyclin-dependent kinases, and the PI3K/AKT/mTOR pathway.

Despite the inherent limitations of this qualitative study, compelling trends within a large database of tumor DNA sequencing results derived from clinically accessible information were identified. A clinical trial assessing the efficacy of berzosertib and irinotecan in TP53 mutant GEA cancers is currently ongoing (NCT03641313) which is the first clinical trial selected for patients based on *TP53* status. The mutational differences of GEAs stratified by *TP53* mutation status as described in this manuscript can be used as a tool when designing and analyzing future pre-clinical and clinical trials to assess the predictive ability of *TP53* mutational status when targeting differentially affected molecular pathways.

## Data Availability

The data underlying this article will be shared on reasonable request to the corresponding author.

## References

[1] Bray F , Ferlay J, Soerjomataram I, Siegel RL, Torre LA, Jemal A. Global cancer statistics 2018: GLOBOCAN estimates of incidence and mortality worldwide for 36 cancers in 185 countries. CA Cancer J Clin. 2018;68(6):394-424. 30207593 10.3322/caac.21492

[2] https://www.cancer.org/research/cancer-facts-statistics/all-cancer-facts-figures/cancer-facts-figures-2021.html.

[3] Thrift AP , Whiteman DC. The incidence of esophageal adenocarcinoma continues to rise: analysis of period and birth cohort effects on recent trends. Ann Oncol. 2012;23(12):3155-3162. 22847812 10.1093/annonc/mds181

[4] GBD 2017 Gastro-oesophageal Reflux Disease Collaborators. The global, regional, and national burden of gastro-oesophageal reflux disease in 195 countries and territories, 1990–2017: a systematic analysis for the Global Burden of Disease Study 2017. Lancet Gastroenterol Hepatol. 2020;5(6):561-581. 32178772 10.1016/S2468-1253(19)30408-X PMC7232025

[5] NCD Risk Factor Collaboration (NCD-RisC). Worldwide trends in body-mass index, underweight, overweight, and obesity from 1975 to 2016: a pooled analysis of 2416 population-based measurement studies in 128.9 million children, adolescents, and adults. Lancet. 2017;390(10113):2627-2642. 29029897 10.1016/S0140-6736(17)32129-3 PMC5735219

[6] Cancer Genome Atlas Research Network. Comprehensive molecular characterization of gastric adenocarcinoma. Nature. 2014;513(7517):202-9. 25079317 10.1038/nature13480 PMC4170219

[7] Tan P , Yeoh KG. Genetics and molecular pathogenesis of gastric adenocarcinoma. Gastroenterology. 2015;149(5):1153-1162.e3. 26073375 10.1053/j.gastro.2015.05.059

[8] Hanazono K , Natsugoe S, Stein HJ, Aikou T, Hoefler H, Siewert JR. Distribution of p53 mutations in esophageal and gastric carcinomas and the relationship with p53 expression. Oncol Rep. 2006;15(4):821-824. 16525665

[9] Mantovani F , Collavin L, Del Sal G. Mutant p53 as a guardian of the cancer cell. Cell Death Differ. 2019;26(2):199-212. 30538286 10.1038/s41418-018-0246-9 PMC6329812

[10] Busuttil RA , Zapparoli GV, Haupt S, et al. Role of p53 in the progression of gastric cancer. Oncotarget. 2014;5(23):12016-12026. 25427447 10.18632/oncotarget.2434 PMC4322971

[11] Cristescu R , Lee J, Nebozhyn M, et al. Molecular analysis of gastric cancer identifies subtypes associated with distinct clinical outcomes. Nat Med. 2015;21(5):449-456. 25894828 10.1038/nm.3850

[12] Park S , Lee J, Kim YH, Park J, Shin JW, Nam S. Clinical relevance and molecular phenotypes in gastric cancer, of TP53 mutations and gene expressions, in combination with other gene mutations. Sci Rep. 2016;6:34822. 27708434 10.1038/srep34822 PMC5052597

[13] Yildirim M , Kaya V, Demirpence O, Gunduz S, Bozcuk H. Prognostic significance of p53 in gastric cancer: a meta-analysis. Asian Pac J Cancer Prev. 2015;16(1):327-332. 25640374 10.7314/apjcp.2015.16.1.327

[14] Xu HY , Xu WL, Wang LQ, Chen MB, Shen HL. Relationship between p53 status and response to chemotherapy in patients with gastric cancer: a meta-analysis. PLoS One. 2014;9(4):e95371. 24740294 10.1371/journal.pone.0095371 PMC3989310

[15] https://www.foundationmedicine.com/test/foundationone-cdx

[16] Bang YJ , Van Cutsem E, Feyereislova A, et al.; ToGA Trial Investigators. Trastuzumab in combination with chemotherapy versus chemotherapy alone for treatment of HER2-positive advanced gastric or gastro-oesophageal junction cancer (ToGA): a phase 3, open-label, randomised controlled trial. Lancet. 2010;376(9742):687-697. 20728210 10.1016/S0140-6736(10)61121-X

[17] Chao J , Fuchs CS, Shitara K, et al. Assessment of pembrolizumab therapy for the treatment of microsatellite instability-high gastric or gastroesophageal junction cancer among patients in the KEYNOTE-059, KEYNOTE-061, and KEYNOTE-062 clinical trials. JAMA Oncol. 2021;7(6):895-902. 33792646 10.1001/jamaoncol.2021.0275 PMC8017478

[18] Sahin U , Türeci Ö, Manikhas G, et al. FAST: a randomised phase II study of zolbetuximab (IMAB362) plus EOX versus EOX alone for first-line treatment of advanced CLDN18.2-positive gastric and gastro-oesophageal adenocarcinoma. Ann Oncol. 2021;32(5):609-619. 33610734 10.1016/j.annonc.2021.02.005

[19] Wainberg ZA , Enzinger PC, Kang YK, et al. Randomized double-blind placebo-controlled phase 2 study of bemarituzumab combined with modified FOLFOX6 (mFOLFOX6) in first-line (1L) treatment of advanced gastric/gastroesophageal junction adenocarcinoma (FIGHT). J Clin Oncol. 2021;39(suppl 3):abstr 160.

[20] Poremba C , Yandell DW, Huang Q, et al. Frequency and spectrum of p53 mutations in gastric cancer—a molecular genetic and immunohistochemical study. Virchows Arch. 1995;426(5):447-455. 7633655 10.1007/BF00193167

[21] Li-Chang HH , Kasaian K, Ng Y, et al. Retrospective review using targeted deep sequencing reveals mutational differences between gastroesophageal junction and gastric carcinomas. BMC Cancer. 2015;15:32. 25656989 10.1186/s12885-015-1021-7 PMC4322811

[22] Fléjou JF , Gratio V, Muzeau F, Hamelin R. p53 abnormalities in adenocarcinoma of the gastric cardia and antrum. Mol Pathol. 1999;52(5):263-268. 10748875 10.1136/mp.52.5.263 PMC395708

[23] Ireland AP , Shibata DK, Chandrasoma P, Lord RV, Peters JH, DeMeester TR. Clinical significance of p53 mutations in adenocarcinoma of the esophagus and cardia. Ann Surg. 2000;231(2):179-187. 10674608 10.1097/00000658-200002000-00005 PMC1420984

[24] Cohen PA , Donini CF, Nguyen NT, Lincet H, Vendrell JA. The dark side of ZNF217, a key regulator of tumorigenesis with powerful biomarker value. Oncotarget. 2015;6(39):41566-41581. 26431164 10.18632/oncotarget.5893 PMC4747174

[25] Pasello G , Agata S, Bonaldi L, et al. DNA copy number alterations correlate with survival of esophageal adenocarcinoma patients. Mod Pathol. 2009;22(1):58-65. 18820669 10.1038/modpathol.2008.150

[26] Ikari N , Serizawa A, Mitani S, Yamamoto M, Furukawa T. Near-comprehensive resequencing of cancer-associated genes in surgically resected metastatic liver tumors of gastric cancer. Am J Pathol. 2019;189(4):784-796. 30703342 10.1016/j.ajpath.2018.12.015

[27] Blanchet A. , et al. Isoforms of the p53 family and gastric cancer: a ménage à trois for an unfinished affair. Cancers (Basel). 2021;13(4):916. 33671606 10.3390/cancers13040916 PMC7926742

[28] Lim BH , Soong R, Grieu F, Robbins PD, House AK, Iacopetta BJ. p53 accumulation and mutation are prognostic indicators of poor survival in human gastric carcinoma. Int J Cancer. 1996;69(3):200-204. 8682588 10.1002/(SICI)1097-0215(19960621)69:3<200::AID-IJC9>3.0.CO;2-3

[29] Deng W , Hao Q, Vadgama J, Wu Y. Wild-type TP53 predicts poor prognosis in patients with gastric cancer. J Cancer Sci Clin Ther. 2021;5(1):134-153. 34950877 10.26502/jcsct.50790107 PMC8694034

[30] Tahara T , Shibata T, Okamoto Y, et al. Mutation spectrum of TP53 gene predicts clinicopathological features and survival of gastric cancer. Oncotarget. 2016;7(27):42252-42260. 27323394 10.18632/oncotarget.9770 PMC5173132

[31] Xu HY , Xu WL, Wang LQ, Chen MB, Shen HL. Relationship ­between p53 status and response to chemotherapy in patients with gastric cancer: a meta-analysis. PLoS One. 2014;9(4):e95371. 24740294 10.1371/journal.pone.0095371 PMC3989310

[32] Hwang HJ , Nam SK, Park H, et al. Prediction of TP53 mutations by p53 immunohistochemistry and their prognostic significance in gastric cancer. J Pathol Transl Med. 2020;54(5):378-386. 32601264 10.4132/jptm.2020.06.01 PMC7483024

[33] Schoop I , Maleki SS, Behrens HM, Krüger S, Haag J, Röcken C. p53 immunostaining cannot be used to predict TP53 mutations in gastric cancer: results from a large Central European cohort. Hum Pathol. 2020;105:53-66. 32971129 10.1016/j.humpath.2020.09.006

[34] Graziano F. , et al. TP53 mutation analysis in gastric cancer and clinical outcomes of patients with metastatic disease treated with Ramucirumab/Paclitaxel or standard chemotherapy. Cancers (Basel). 2020;12(8):2049. 10.3390/cancers12082049 PMC7465166 32722340

[35] Li L , Li M, Wang X. Cancer type-dependent correlations between TP53 mutations and antitumor immunity. DNA Repair (Amst). 2020;88:102785. 32007736 10.1016/j.dnarep.2020.102785

[36] Liu YZ , et al. Olaparib plus paclitaxel sensitivity in biomarker subgroups of gastric cancer. Ann Oncol. 2018;29(suppl_8):814-857.

[37] Gao JP , Xu W, Liu WT, Yan M, Zhu ZG. Tumor heterogeneity of gastric cancer: from the perspective of tumor-initiating cell. World J Gastroenterol. 2018;24(24):2567-2581. 29962814 10.3748/wjg.v24.i24.2567 PMC6021770

